# Electro-Active Paper as a Flexible Mechanical Sensor, Actuator and Energy Harvesting Transducer: A Review

**DOI:** 10.3390/s18103474

**Published:** 2018-10-15

**Authors:** Asif Khan, Faisal Raza Khan, Heung Soo Kim

**Affiliations:** Department of Mechanical, Robotics and Energy Engineering, Dongguk University-Seoul, 30 Pil-dong 1 Gil, Jung-gu, Seoul 04620, Korea; khanuet11@gmail.com (A.K.); faisalrazakhan1991@gmail.com (F.R.K.)

**Keywords:** electro-active paper (EAPap), flexible smart material, characterization

## Abstract

Electro-active paper (EAPap) is a cellulose-based smart material that has shown promising results in a variety of smart applications (e.g., vibration sensor, piezo-speaker, bending actuator) with the merits of being flexible, lightweight, fracture tolerant, biodegradable, naturally abundant, cheap, biocompatible, and with the ability to form hybrid nanocomposites. This paper presents a review of the characterization and application of EAPap as a flexible mechanical vibration/strain sensor, bending actuator, and vibration energy harvester. The working mechanism of EAPap is explained along with the various parameters and factors that influence the sensing, actuation, and energy harvesting capabilities of EAPap. Although the piezoelectricity of EAPap is comparable to that of commercially available polyvinylidene fluoride (PVDF), EAPap has the preferable merits in terms of natural abundance and ample capacity of chemical modification. The article would provide guidelines for the characterization and application of EAPap in mechanical sensing, actuation, and vibration energy scavenging, along with the possible limitations and future research prospects.

## 1. Introduction

Smart or functional materials are the kind of materials that respond to external stimuli in a controlled fashion by altering one or more of their inherent properties [1,2]. Since the discovery of the piezoelectric effect by the Curie brothers in 1880 [3], a lot of research efforts have been devoted to the development [4,5,6] and industrial application [7,8] of smart materials. Examples of some well-known smart materials reported in the literature are piezoceramic [9], piezo polymers [10], shape memory alloys [11], electroactive polymers [12], electrorheological and magnetorheological fluids [13], and covalent adaptive network polymers [6], among others. Piezoelectric materials have shown promising results in mechanical vibration sensing [14,15,16], actuation [17,18], and energy transduction applications [19,20]. Piezoelectric ceramics (e.g., lead zirconate titanate (PZT)) and piezoelectric polymers (polyvinylidene fluoride (PVDF)) are the most commonly used, commercially available piezoelectric materials. However, PZT and PVDF suffer from the following limitations: the fabrication of PZTs requires the toxic material of lead oxide to be produced, whereas PVDF is a petroleum-based polymer, and suffers from extreme temperature fluctuations and various types of radiation. Hence, there exists a need for environment friendly and natural resource-based renewable piezoelectric materials.

Electro-active paper (EAPap), discovered by Kim et al. [21], is a cellulose-based smart material that has shown promising results as piezoelectric actuator, mechanical vibration and strain sensor, vibration energy harvesting transducer, flexible speaker, paper transistor, micro-flying object, and MEMS/NEMS device [22]. The raw material of EAPap is cellulose that is a nontoxic, colorless, and odorless solid with a per year production of 100 billion tons on Earth from the natural resources of plants, cotton, and seaweed [5,23]. Some promising advantages of EAPap as a smart material are its flexibility, transparency, low cost, high mechanical strength, renewable nature, large displacement output, low actuation voltage, biodegradable characteristics, and dryness [21,24,25]. The electromechanical properties of EAPap without any additive are comparable to that of PVDF, and can be substantially improved by wet drawing [26], coating of nanolayer of zinc oxide [27], and the hybridization of cellulose with nanocomposites [22]. EAPap is fabricated in the form of thin film with a thickness of (20–30) μm and electrodes coated on both sides. The flexible nature of EAPap allows it to be attached to surfaces of different geometric configurations, and does not influence the resonant frequencies of the host structure, due its ultra-light weight nature. Also, the flexibility of EAPap allows it to be used in harsh vibration, where PZTs cannot be employed, due to their brittleness.

This review article places emphasis on the characterization of cellulose-based electroactive paper (EAPap) as mechanical vibration/strain sensor, actuator, and energy harvesting transducer. The actuation, sensing, and energy transduction mechanisms of EAPap are briefly summarized in a short initial section. The contents of the sections that follow are dedicated to a thorough and up-to-date discussion on the characterization and application of EAPap for flexible piezoelectric actuation, vibration or strain sensing, and mechanical vibration energy harvesting, along with possible challenges and limitations. Various parameters, such as fiber orientation, type of electrode, power, and surrounding noise, that affect the performance of EAPap are identified and discussed. The article provides a comprehensive review of the characterization and application of EAPap for mechanical actuation, sensing, and vibration energy harvesting.

## 2. Working Principle of EAPap

**Figure 1:**
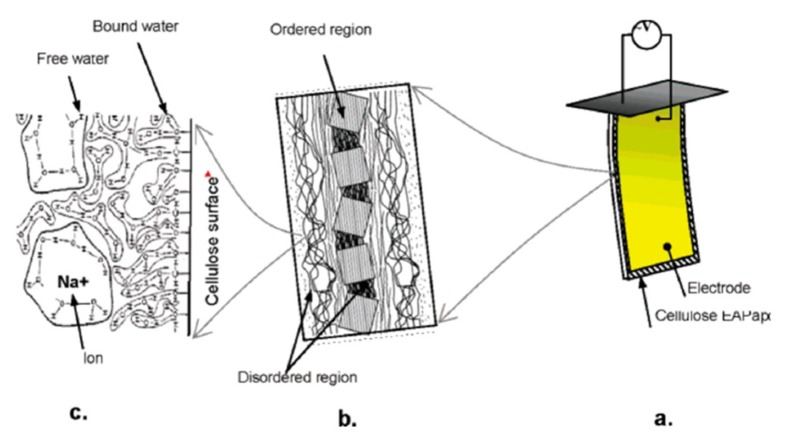
Concept of electro-active paper (EAPap) actuator: (**a**) EAPap is made from cellulose paper on which gold electrodes are deposited on both sides; (**b**) cellulose microfibril has ordered crystalline regions and disordered regions; (**c**) water molecules are bonded with hydroxyls on the cellulose surface (bound water) or clustered in free (free water) [21].

Cellulose-based EAPap has shown promising results as flexible sensor, actuator, and energy harvesting transducer [28]. From the morphological point of view, EAPap is a sheet of regenerated cellulose that consists of ordered and disordered regions. The ordered regions are characterized by crystalline and amorphous structure, whereas the disordered regions are characterized by the presence of water molecules and sodium ions that are injected during the fabrication process of EAPap. Also, in the disordered region, there exists a lot of hydroxyl groups around the disordered chains of cellulose. The water molecules may be attached to the hydroxyl group or present as free water, as shown in the conceptual configuration of EAPap in Figure 1c.

In the disordered region, the water molecules can move freely, as well as interact with the sodium ions. The crystalline structure in the ordered region of regenerated cellulose is responsible for the piezoelectric effect, whereas the interaction of hydroxyl groups with the water molecules and sodium ions results in an ion migration effect. It is believed that the combined effect of the inherent piezoelectricity in the ordered regions and ion migration in the disordered region is responsible for the working of cellulose EAPap as a smart material. In the form a piezoelectric actuator, when an electric field is applied to EAPap, a bending phenomenon is observed as a result of the volumetric change of the EAPap that is caused by the movement of sodium ions surrounded by water molecules towards the anode of the external electric field [21]. 

In general, the polarizability of cellulose EAPap is attributed to the electronic contribution from the displacement of the electron shell relative to nucleus, ionic contribution from the displacement of charged ion relative to other ions, and permanent molecular dipole moments of the hydroxyl and carboxyl groups. In particular, the hydrogen bonding of cellulose chains in the disordered regions (Figure 1c) of EAPap results in many localized states that dominate the charge transfer process upon the release or excitation of charge carriers in these states. Hence, the permanent polarization and subsequent piezoelectric behavior of EAPap is observed due to the disordered regions that stabilize the dipoles and lead to permanent polarization. The piezoelectric effects of EAPap make it a promising candidate as a flexible piezoelectric sensor, artificial muscle, and energy harvesting transducer [29,30,31].

## 3. EAPap as a Flexible Vibration/Strain Sensor

Cellulose-based electro-active paper has shown promising results as a flexible vibration and strain sensor in measuring the dynamic characteristics of vibrating structures. Some advantages of EAPap as a vibration sensor over other piezoelectric materials are its eco-friendly behavior, flexibility, light weight, natural abundance, low cost, and ease of manufacturing [21,32], among others. From a structural viewpoint, the piezoelectricity of EAPap is associated with the dipolar orientation and monoclinic structure of cellulose. As a flexible piezoelectric sensor, EAPap generates surface charge as a response to mechanical deformation. This phenomenon is called direct piezoelectricity, and is governed by the mathematical expression of Equation (1)

(1) $$D_{i}=d_{ij}c_{jk}\varepsilon_{k}+k_{im}E_{m}\;i,j=1,\;2,\;3\;$$

where $\varepsilon_{k},\;D_{i}$, and $E_{m}$ denote the strain tensor, dielectric displacement vector, and electric field vector, respectively. The terms $d_{ij}$, $c_{jk}$, and $k_{im}$ refer to matrices of piezoelectric, elastic stiffness, and dielectric permittivity constants, respectively.

When EAPap is used as sensor, no electric field is applied ($E_{m}=0$) and the charge of interest ($D_{3}$) is collected through the electrodes on upper and lower surfaces of EAPap. Equation (1) simplifies to the expression of Equation (2):

(2) $$D_{3}=d_{31}c_{1k}\varepsilon_{k}$$

The piezoelectric charge constant of EAPap is determined experimentally from the expression of Equation (3) [33]:

(3) $$d_{31}={\left(\frac{\partial D_{3}}{\partial T1}\right)}_{E}=\frac{\mathrm{induced}\;\mathrm{charge}\;\mathrm{per}\;\mathrm{unit}\;\mathrm{electrode}\;\mathrm{area}}{\mathrm{Applied}\;\mathrm{in}-\mathrm{plane}\;\mathrm{normal}\;\mathrm{stress}\;}\;(C/N)$$

where the subscript *E* denotes the constant electric field imposed during the pulling test for determining piezoelectric charge constant.

**Figure 2:**
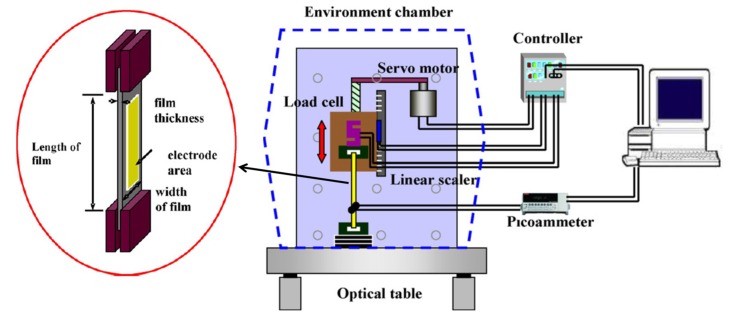
Schematic of piezoelectric charge constant measurement based on direct piezoelectricity [31].

Figure 2 shows a schematic of the process of experimentally determining the piezoelectric charge constant using Equation (3) [31].

**Figure 3:**
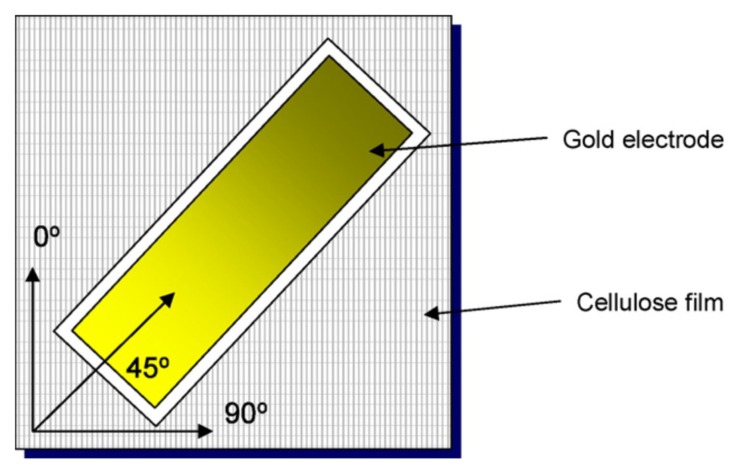
Orientation of cellulose film and schematic of EAPap [31].

The effect of directionality on the piezoelectric charge constant of EAPap was investigated by considering three different orientations, as shown in Figure 3. 

Table 1 shows the piezoelectric charge constant of EAPap for different orientation within the elastic limit.

Kim et al. [34] investigated the possibility of EAPap as a vibration sensor. The average capacitance and relative permittivity of EAPap were found to be the same as those of commercially available synthetic PVDF. It was found that the sensing capabilities of EAPap are not useful in the presence of ambient and power noise; however, grounding and shielding of EAPap dramatically reduces the effect of noise, and EAPap can correctly capture the dynamic response characteristics (i.e., natural frequencies) of the beam without any charge amplifier. Furthermore, the grounded and shielded EAPap showed better performance as a vibration sensor than the accelerometer, as the EAPap clearly captured two twisting modes that were not clearly obtained by the accelerometer. Abas et al. [35] characterized EAPap as a vibration sensor by impact testing and random excitation. The performance of EAPap as a vibration sensor was compared with that of PVDF. The dynamic response in the frequency domain revealed that EAPap sensor has good sensitivity to a lower level of strain and low frequency vibration; however, the usefulness of EAPap as a sensor in the high-frequency range is limited due to power noise. Also, the comparison of the time domain response of the EAPap and PVDF sensors to the same input revealed that EAPap is more sensitive to ambient and power noise than PVDF.

**Figure 4:**
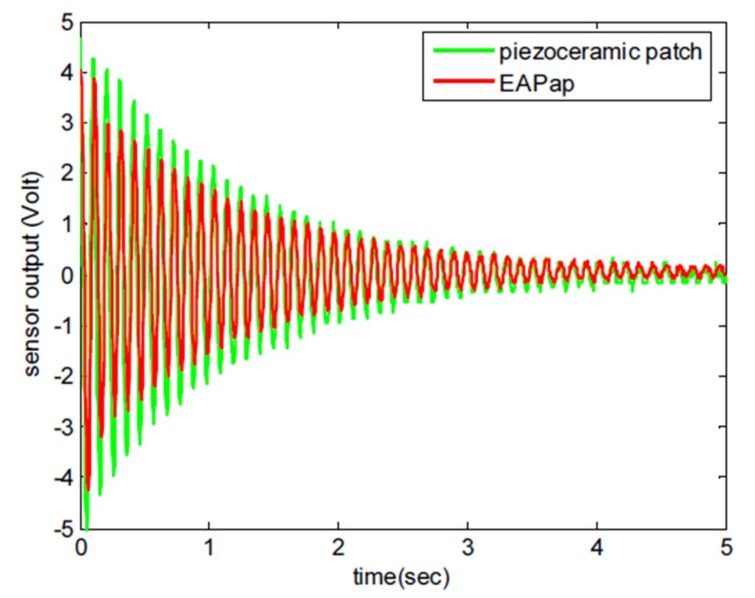
Impulsive response of the beam measured by EAPap and piezoceramic patch [29].

Kim et al. [29] investigated the possibility of using EAPap as a piezoelectric sensor by studying the vibration control of a cantilevered beam made of aluminum. A PID-based feedback controller was employed to suppress the vibration of the beam by minimizing the output of the EAPap sensor, which was considered as a position error of the cantilevered beam. The open and closed loop performance of the controller revealed that EAPap has great potential as a piezoelectric vibration sensor. Also, comparison of the impulse responses as measured by EAPap and PZT-5H showed that EAPap has sensing capability similar to that of the piezoceramic patch (PZT-5H) for low frequency vibration as shown in Figure 4. 

Lee et al. [36] studied the direct piezoelectricity of EAPap by subjecting the EAPap to in-plane normal static/dynamic load, and measuring the induced charge and voltage during a pull test. It was found that the piezoelectric charge constant of dimethylacetamide (DMAC) EAPap is dependent on the material orientation (0, 45, 90)° and mechanical drawing ratio (*D_r_*), as shown in Table 2.

Mun el al. [37] characterized the strain sensing behaviors of a flexible and transparent cellulose film coated with silver nanowires. Silver nanowires (AgNW) were sprayed on the dried cellulose film via a spray layer-by-layer technique; it was found that the sheet resistance decreases with an increase in the concentration of AgNW solution, and a trade-off exists between the transmittance and resistance of AgNW-coated cellulose films. The mechanical, electrical, and strain-sensing characteristic of the AgNW-coated cellulose films were quantified in the stretching and bending modes under cyclic loadings.

**Figure 5:**
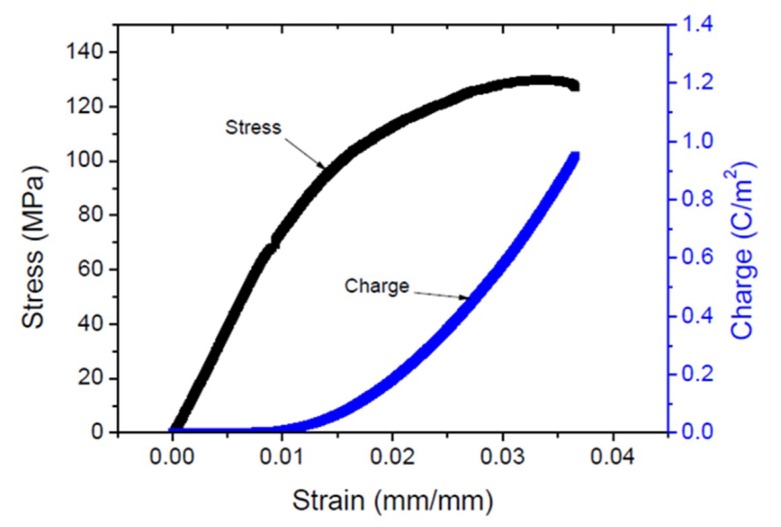
Stress–strain and charge curves of cellulose ZnO hybrid nanocomposite (CEZOHN) [38].

Ko et al. [38] investigated the strain sensing behavior of cellulose ZnO hybrid nanocomposite (CEZOHN) in bending and longitudinal stretching modes. Figure 5 shows the stress–strain curve and induced charge curve of the CEZOHN, while Table 3 compares its electromechanical properties with bare and aligned cellulose [39].

**Figure 6:**
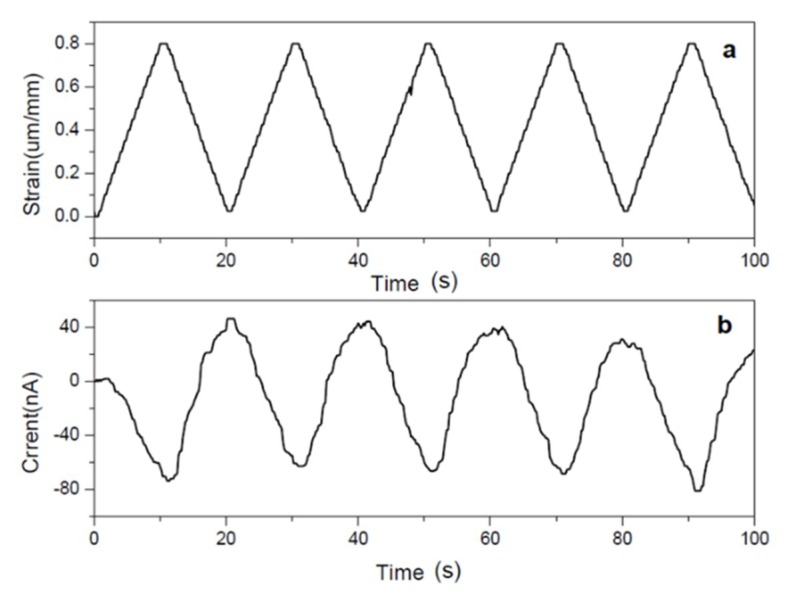
Stretching mode strain sensor: (**a**) stretching strain and (**b**) sensor signal [38].

Table 3 shows that ZnO did not affect the mechanical rigidity of the cellulose; however, the piezoelectric charge constant was increased 30 times compared with bare cellulose, and 6 times compared with aligned cellulose. The stretching test showed that the induced current signal of CEZOHN closely followed the applied stretching strain, as shown in Figure 6.

## 4. EAPap as a Flexible Actuator

Among the various available electro-active polymers (EAPs), conductive polymers [40], ionic polymer metal composite [41], dielectric elastomers [42], gel-polymers [43], and so on, EAPap has been spotlighted as a promising biomimetic actuator. Some noticeable characteristics of EAPap as an actuator are its large displacement output, dryness, flexibility, low actuation voltage, biodegradability, low power consumption, and light weight nature [21,44,45]. Various parameters that influence the performance of EAPap as an actuator are its fabrication processes, type of solvent in which cellulose pulp is dissolved, deposition of electrode and the geometry of the pattern of the electrode, mechanical stretching, and environmental conditions of temperature and humidity. A comprehensive literature review on various aspects of EAPap, its hybrid nanocomposites, and applications up to 2016 can be referred to in a previous paper [22] from the same authors. In this section of the paper, we review some fundamental aspects of EAPap as an actuator for mechanical applications.

As a flexible piezoelectric actuator, EAPap works on the principle of converse piezoelectricity, where an external electric field induces mechanical strain in EAPap. Yun et al. [46] experimentally quantified the converse piezoelectric charge constant using Equation (4).

(4) d31=(∂S1∂E3)T=induced in-Plane strainApplied voltage/thickness [mV]

where *S* denotes the in-plane induced strain, *E* is the externally applied electric field, and the subscript *T* denotes boundary condition of constant stress during the applied electric field. The subscripts 1 and 3 refer to the in-plane and out-of-plane orientation of the sample, respectively. The *d*_33_ mode of the piezoelectric charge constant can be quantified from the general relation of Equation (5) for converse piezoelectric effect [33].

(5) d=(∂S∂E)T=induced in-Plane strainApplied voltage/thickness [mV]

**Figure 7:**
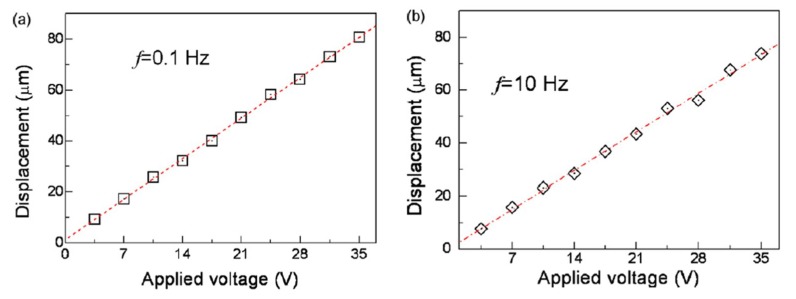
Plots of the bending displacement versus the applied voltage (Vamp) at the frequencies of (**a**) *f* = 0.1 Hz and (**b**) *f* = 10 Hz [47].

Yun et al. [47] investigated the performance of a thin stretched EAPap film in the form of unimorph type and stacked type actuators as functions of frequency and applied electric field. The bending displacement of the stacked actuator (*d*_33_ mode) was found to be strongly dependent on frequency of operation such that an increasing operating frequency caused an exponential decrement in the piezoelectric charge constant. For a unimorph actuator (*d*_31_ mode), the bending displacement was observed to be a linear function of the applied voltage and almost independent of the operating frequency, as shown in Figure 7.

The performance of the EAPap not only depends on the material fiber orientation, but also on the electrode pattern, which is usually deposited as a thin layer on both sides of the EAPap. Ridley et al. [48] studied pattern electrode of fishbone geometry, and compared it with the previous research on rectangular electrode pattern [49]. Both types of electrode pattern geometries were compared in terms of bending displacement, resonance frequency, and electrical power consumption. The maximum actuator force and the corresponding maximum mechanical power output of the EAPap actuators were obtained from the bending displacement via Equations (6) and (7), respectively.

(6) $$F=\frac{3EI\delta }{L^{3}}=\frac{Ebh^{3}\delta }{4L^{3}}$$

(7) $$P_{\max}=2\pi \delta fF=\pi \frac{fEbh^{3}\delta^{2}}{2L^{3}}$$

where *F* and *P*_max_ are the maximum actuator force and maximum mechanical power output, respectively. The term δ refers to the measured bending displacement of EAPap, *I* and *E* denote the moment of inertia and Young’s modulus of the EAPap, respectively. The quantities *b*, *h*, and *L* are the width, thickness, and length of the EAPap sample, respectively.

The performance of the rectangular and fishbone electrodes were compared in terms of bending displacement, resonance frequencies, electrical power consumptions, mechanical power output, and actuator efficiency on two samples of EAPap, namely DCell and cellophane; as shown in Table 4.

Although the fishbone pattern of electrodes showed 30–60% improvement in the actuator efficiency (mechanical power output/electrical power consumption), its electrodes were more severely damaged than rectangular electrodes as a result of more electric field concentration around the ‘*fingers*’ of the fishbone pattern. 

**Figure 8:**
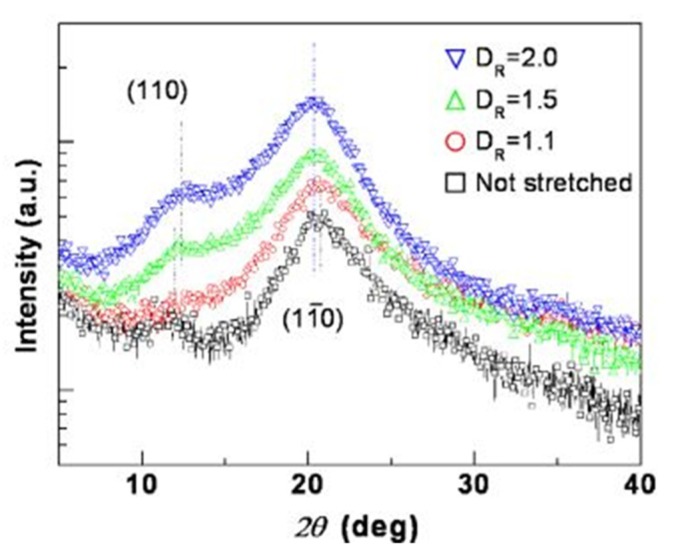
X-ray diffraction data of the stretched cellulose film with different stretching ratios [50].

The mechanical stretching effect also affects the performance of the EAPap. Kim et al. [50] observed the lattice elongation of cellulose fibrils due to in-plane tensile stress along the stretching direction by X-ray diffraction method. They stretched the cured cellulose film at different ratios (*D_R_* = 1.1, 1.5, and 2.0). Figure 8 shows a comparison of the measured wide X-ray diffraction data of the stretched EAPap film with the non-stretched EAPap for 2θ angle from (5 to 40)°.

**Figure 9:**
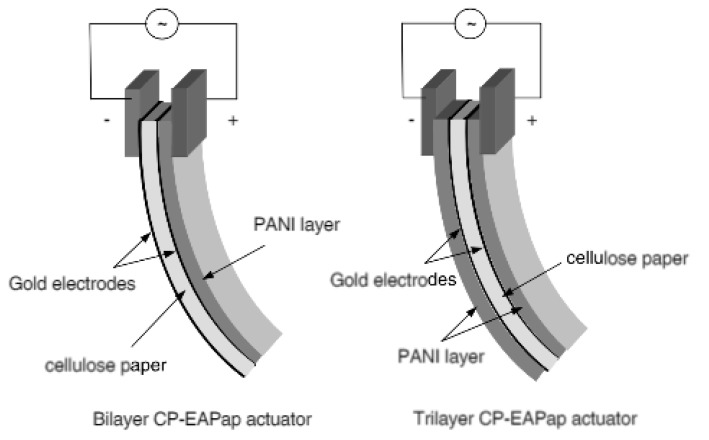
Schematic of bi-layer and tri-layer CP–EAPap actuators [51]. (PANI-polyaniline).

Two peaks were observed for the stretched EAPap, as well as for the non-stretched one, that is, 110 and $1\bar{1}0$, respectively. The first peak (110) for the non-stretched EAPap was observed at ~12.2°, which was slightly lower than any of the three stretched EAPaps (~12.5°). The second peak was observed at 20.6° for both the stretched and non-stretched EAPap; however, for the stretched EAPap with a *D_R_* of 2.0, the 1$\bar{1}$0 peak became dominant, which confirmed the improvement of the EAPap as an actuator in the stretching direction of the sample. The performance of the EAPap is also maximized with a conductive coating on its surface. Kim et al. [51] electrochemically deposited conductive polyaniline on a cellulose paper. The performance of the coated EAPap increased three times compared with the non-coated EAPap. Furthermore, it was observed that in terms of bending displacement, a tri-layer EAPap actuator performed better than a bi-layered actuator. Figure 9 shows a schematic of the bilayer and tri-layer EAPap actuators coated with conductive polymers (CP–EAPap actuators).

**Figure 10:**
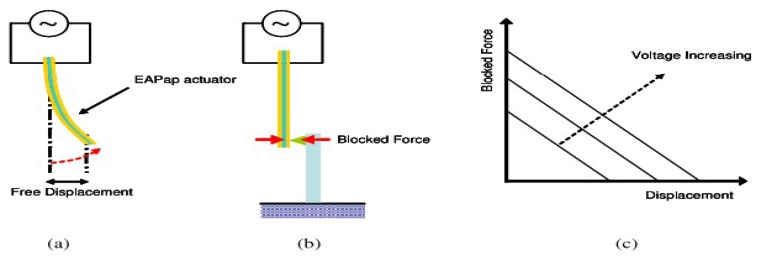
Schematic diagram of performance parameters: (**a**) free displacement, (**b**) blocked force, (**c**) force–displacement relation with increasing voltage [52].

Various test methods have also been developed to measure the pertinent performance parameters of EAPap as a flexible actuator. Of these test methods, tip deflection, radius of curvature, and blocked force are the important parameters that measure the performance of EAPap as a bending actuator [52]. Figure 10 shows the three types of performance parameters, that is, (a) free displacement, (b) blocked force, and (c) blocked force versus displacement relation with increasing voltage.

**Figure 11:**
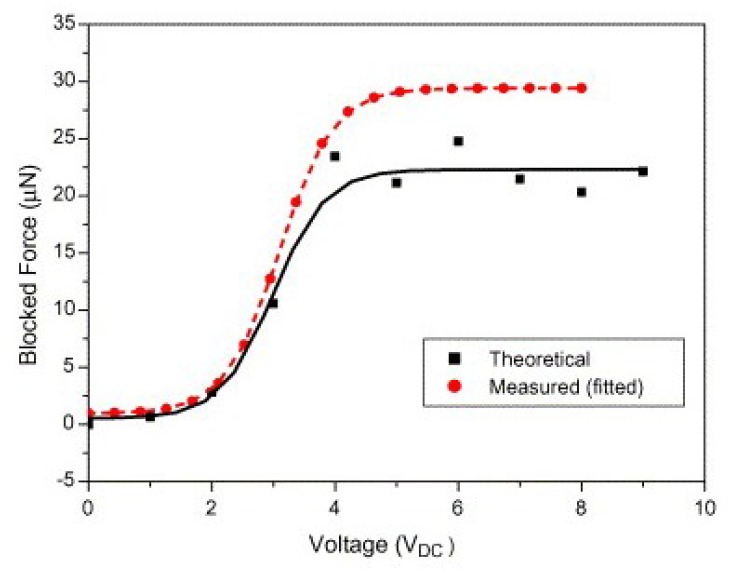
Comparison of measured and calculated blocked forces on DC electric field [53].

Kim et al. [53] measured the blocked force of EAPap bending actuator using a microbalance, and compared it with a cantilever beam model blocked force. Figure 11 shows the comparison of measured blocked force with the theoretical blocked force calculated from the relation of Equation (8):

(8) $$P=\frac{3EI\delta }{L^{3}}=24.9\;\mu N$$

where *P* is the theoretical blocked force, *E* is the elastic modulus of EAPap, *I* is the moment of inertia, *δ* is the displacement, and *L* is the length of the EAPap.

Figure 11 shows that the theoretical and measured blocked force have minute uncertainty up to a DC voltage of 4 V. After 4 V, the measured blocked force has a deviation of 5 μN, which was considered to be in the error range. The theoretical and the measured blocked force followed the same trend against DC voltage, which confirmed the high accuracy of the measured blocked force while using a micro-balance.

**Figure 12:**
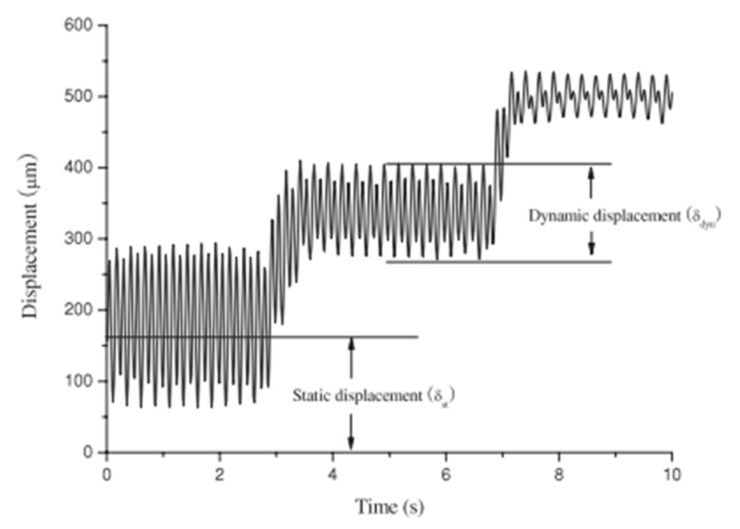
Static and dynamic tip deflection of the force transducer under 350 V mm^−1^ and 80% relative humidity condition [52].

Yun et al. [52] measured the blocked force of the EAPap actuator by a custom-built force transducer. The aim of this study was to measure the blocked force of an EAPap actuator through AC actuation, rather than DC actuation. Figure 12 shows the static and dynamic tip deflection of the force transducer under 350 V mm^−1^ and 80% relative humidity condition.

Figure 12 shows that after a certain period of time, stable dynamic tip deflection of the force transducer is obtained. The transducer force was defined as the sum of the static and dynamic deflections, as given by Equation (9):

(9) $$F=k\left(\delta_{st}+\delta_{dyn}\right)$$

**Figure 13:**
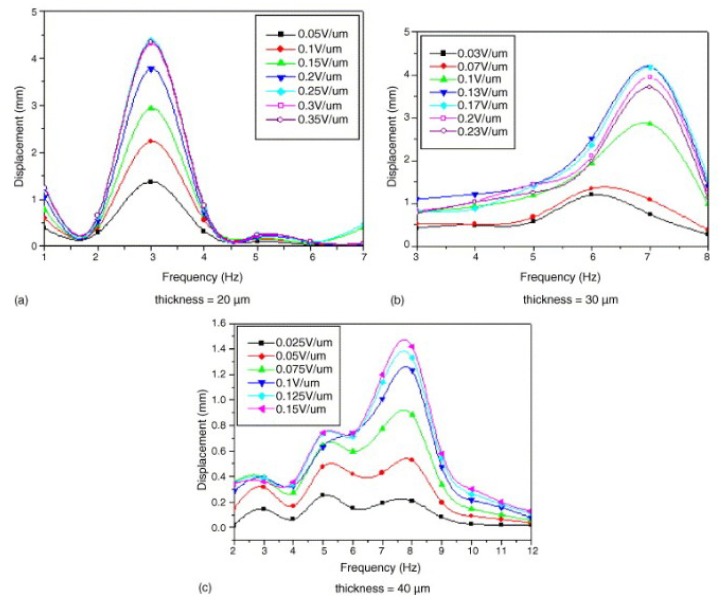
(**a**–**c**) Tip displacements of EAPap actuators with thickness variation [32].

Another physical property that affects the performance of EAPap as a flexible actuator is its thickness. Yun et al. [32] studied three actuators of thicknesses (20, 30, and 40 μm) in terms of tip displacement, blocked force, electrical power consumption, and efficiency, and found that the mechanical properties drastically increased with increasing thickness. Figure 13 shows the tip displacements of three EAPap actuators of different thickness (20, 30, and 40 μm) with voltage and frequency variations.

Herein, it is observed that the resonance frequency corresponding to maximum displacement has increased from 3 to 7 Hz and 8 Hz as the thickness increases from 20 to 30 μm and 40 μm, respectively. Furthermore, the maximum displacement is decreasing with an increase in the thickness of the sample. The EAPap sample of 30 μm thickness showed maximum mechanical power output among the three samples.

## 5. EAPap as Flexible Vibration Energy Harvesting Transducer

The autonomous operation of low-power microscale electronic devices (e.g., wireless sensors, implantable medical devices) mandates the extraction of power from minute but pervasive sources, such as mechanical vibration [20,54,55,56], light [57], heat [58,59], radio frequency (RF) [60,61], and raindrops [62,63], among others. Ambient vibration is an appealing energy source for micro energy transduction because of its abundance [64,65]. Piezoelectric materials have been extensively used for energy harvesting from mechanical vibration [20,66,67]. In general, piezoceramics, such as lead zirconated titanate (PZT), are the most commonly employed materials for harvesting energy from mechanical vibration [68,69,70,71]. However, the brittle nature of PZTs hinder their application for energy harvesting from harsh vibration [68]; and alternative materials, such as PVDF [72,73], ZnO piezoelectric thin films [74], Nafion [75], electro-active polymer (EAP) [76,77], and EAPap [25], are receiving attention for energy transduction from mechanical vibration. In this section, the use of EAPap as energy harvesting transducer is reviewed. Some noticeable advantages of EAPap as an energy harvesting transducer are its light weight nature, natural abundance, low cost, eco-friendliness, and fracture tolerance [22,78], among others.

Abas et al. [25] studied the possibility of EAPap as energy scavenging transducer, and found that EAPap can be employed as a flexible vibration energy harvesting transducer. Furthermore, the voltage output from EAPap energy harvester was found to be dependent on the area of electrodes deposited on EAPap. Hosseini et al. [79] presented an analytical model for calculating the energy generated from the vibration of a cantilever substrate partially covered by EAPap material. Table 5 shows that the analytical model was found to be in good agreement with the experimental results.

**Figure 14:**
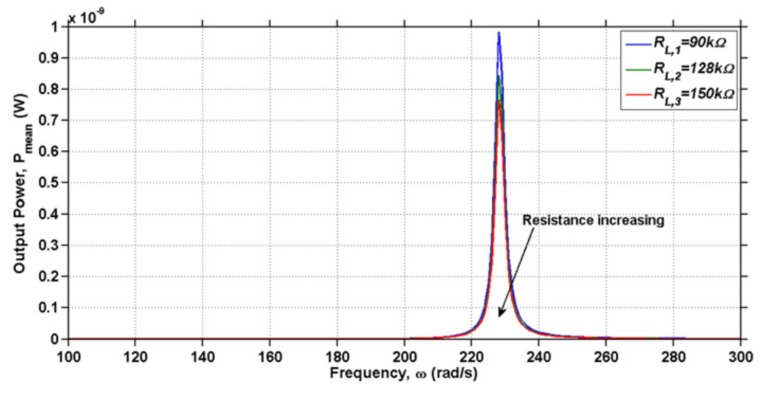
Mean power output of the EAPap-based energy harvester for a range of frequencies with different external resistive loads [79].

**Figure 15:**
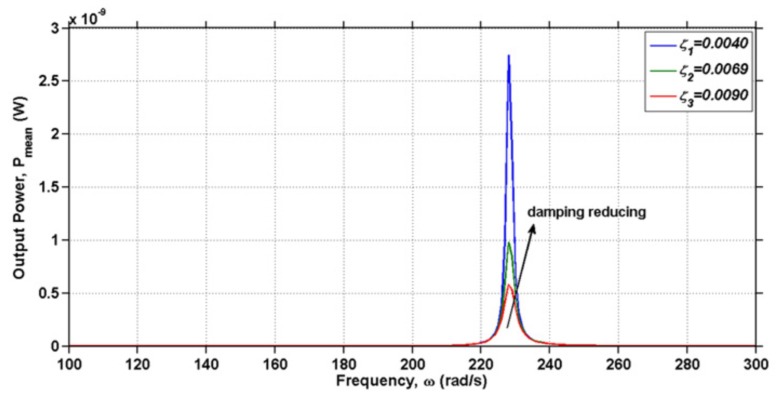
Mean power output of the EAPap-based energy harvester for a range of frequencies in different damping ratios [79].

The harvested current, power, and voltage were found to be significant around the resonance frequency of the structure, and the value of load resistance (RL) and damping ratios were identified as important parameters that influence the harvested power, as shown in Figure 14 and Figure 15.

**Figure 16:**
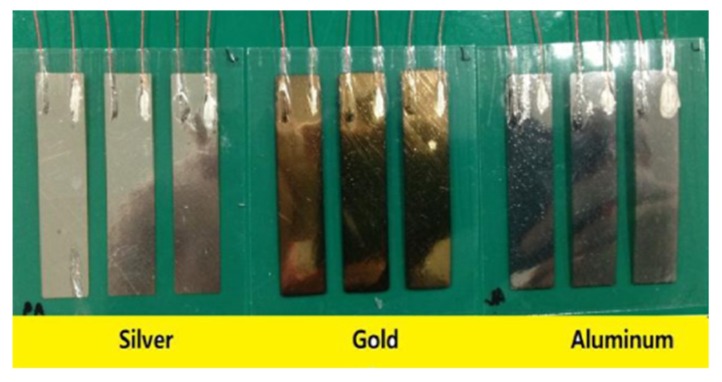
Fabricated gold, silver and aluminum electrode electroactive paper specimens [81].

In an experimental study of vibrational energy harvesting from EAPap [80], it was found that the frequency response functions (FRFs) of voltage and current show a monotonic trend with an increase of the load resistor from 100 kΩ to 1 MΩ; however, the current FRFs showed an opposite trend from voltage FRFs. Also, the output power FRFs did not show a monotonic trend. Abas et al. [81] studied the effect of electrodes of different materials on the energy harvesting capabilities of EAPap. Figure 16 shows that three different specimens of EAPap were prepared with electrodes of gold, silver, and aluminum, respectively.

**Figure 17:**
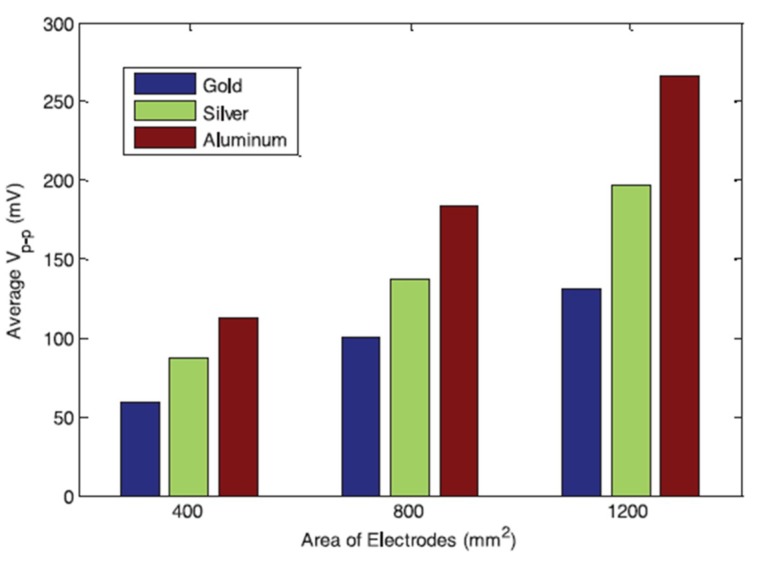
Average peak to peak open circuit voltage output for 400, 800, and 1200 mm^2^ gold, silver, and aluminum electrode-coated EAPap [81].

**Figure 18:**
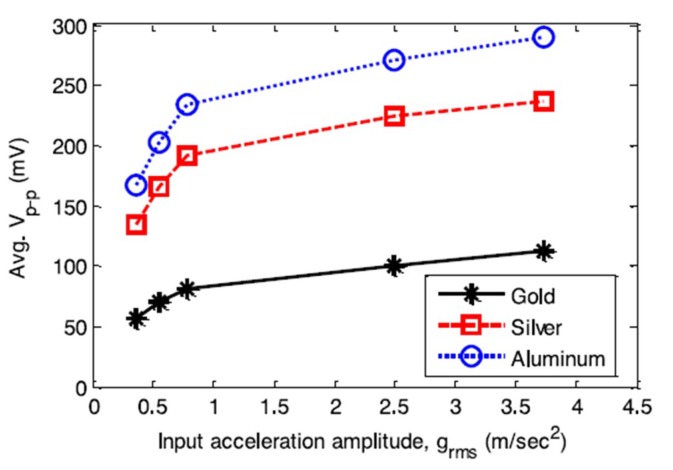
Variation of the average V_p-p_ against the input acceleration amplitude (g_rms_) for three parallel connected electrodes with an area of 1200 mm^2^ [81].

Figure 17 shows that for different area of electrodes, the EAPap specimen with aluminum electrodes was found to produce the largest open circuit voltage, compared with the EAPap specimens of gold and silver electrodes. Figure 18 shows that although the peak-to-peak voltage output from all the specimens increased as the amplitude of the input acceleration to the cantilever vibration bender was increased, the rate of increase of the output voltage was identified to decrease with increasing input acceleration.

**Figure 19:**
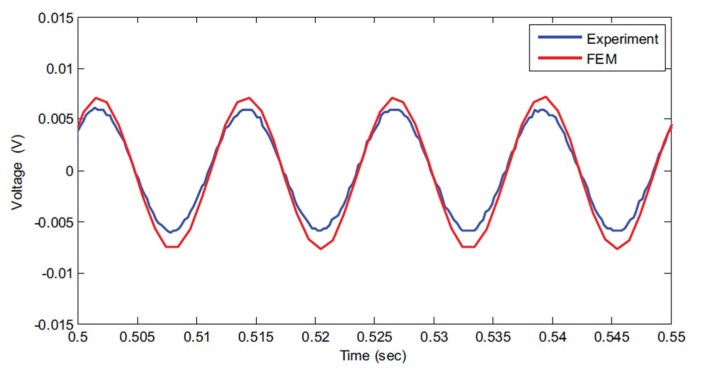
Voltage output produced by EH100 (energy harvester of length 100 mm) when excited by a sinusoidal force at 18 mm from the free end [82]. FEM—finite element model.

Abas et al. [82] developed a coupled-field finite element model (FEM) of EAPap energy harvester, and verified the results experimentally. Figure 19 compares the experimental and FEM results of the voltage output from an aluminum cantilever bender bonded with EAPap.

Mun et al. [27] uniformly coated a nanolayer of zinc oxide on the surface of a regenerated cellulose film, and found drastic improvement in the electromechanical properties of the zinc oxide nanocoated cellulose film (ZONCE), as shown in Table 6.

**Figure 20:**
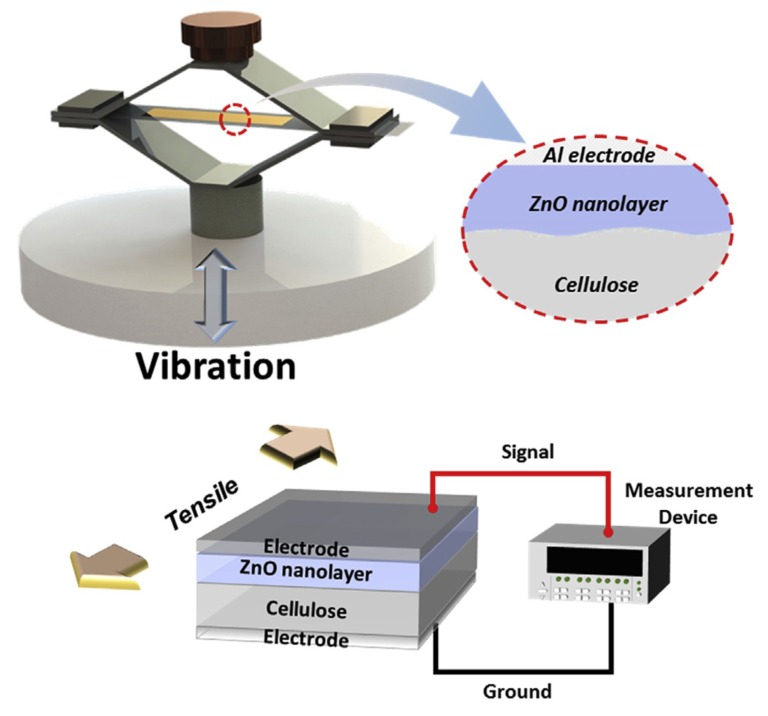
Cymbal type vibration energy harvester [27].

Herein, the piezoelectric charge constant of ZONCE is observed to be 3.5 and 5 times higher than the cellulose EAPap and PVDF, respectively. Figure 20 shows that the potential application of ZONCE for vibration energy harvesting was also demonstrated in a cymbal type vibration energy harvester. The peak-to-peak force (F_p-p_), output voltage (V_p-p_), induced current (I_p-p_), and power output (W_p-p_) of ZONCE were found to be comparable to those of nanogenerators made of ZnO nanorodes and nanowires.

Im et al. [83] investigated the effect of width reduction on a cantilever type EAPap energy harvester, and found that the widthwise split of EAPap and the cantilever beam resulted in more electrical energy than a single beam of the same total width.

## 6. Conclusions

This review article summarizes the characterization and applications of cellulose-based electro-active paper (EAPap) as a flexible mechanical vibration/strain sensor, actuator, and vibration energy scavenger. The experimental process of determining the piezoelectric charge constant of EAPap has been discussed. The piezoelectric charge constant of EAPap is dependent on the orientation of fibers of cellulose, and has the highest value for the fiber orientation of 45°. The average capacitance and relative permittivity of EAPap have been found to be similar to that of PVDF. Although the EAPap sensor has shown better performance than the accelerometer in measuring the lower modes of vibration, its sensing capabilities are strongly influenced by the ambient and power noise, which can be dealt with via grounding and shielding of the EAPap sensor. EAPap has also shown promising results as a strain sensor. The electromechanical properties and strain sensing capabilities of EAPap have been tremendously enhanced by chemically growing ZnO nanorodes on cellulose. The important parameters that influence the harvested current, power, and voltage output from the EAPap energy scavenging transducer are materials of the electrodes, area of the electrodes, load resistance, and damping of the vibration bender. Some noticeable parameters that affect the performance of EAPap as an actuator are the pattern of electrodes (rectangular, fishbone etc.), mechanical stretching, conductive coating, environmental conditions of temperature and humidity, and thickness of the EAPap. Although EAPap has the obvious advantages of biocompatibility, natural abundance, recyclability, eco-friendliness, flexibility, fracture tolerance, ease of manufacturing, and light weight, further research efforts are needed to overcome the possible challenges associated with the real world applications of EAPap, such as the following:

1. Its stability in harsh environment of high temperature and relative humidity.
2. The decrement in the Young’s modulus of EAPap with an increment in the relative humidity due to hydrophilic nature of cellulose.
3. The decrease in the dielectric constant and subsequent actuation behavior of EAPap due to the vaporization of water molecules at elevated temperatures.
4. Poor performance of EAPap vibration sensor in the presence of power and surrounding noise.

## Figures and Tables

**Table 1:** Piezoelectric charge constants of electro-active paper (EAPap) according to material orientation [31].

Orientation	d_31_ (pC/N)
0°	8.01
45°	28.2
90°	23.4

**Table 2:** Piezoelectric charge constant of dimethylacetamide (DMAC) EAPap [36].

Drawing Ratio (*D_r_*)	Piezoelectric Charge Constant (pC/N)		
	0°	45°	90°
1.0	-	0.40–0.41	-
1.5	2.5	3.1	0.65
2.0	5.2	7.3	0.39

**Table 3:** Comparison of Young’s modulus and piezoelectric charge constant of cellulose and cellulose ZnO hybrid nanocomposite (CEZOHN) [38,39].

Samples	Young’s Modulus (GPA)	Piezoelectric Charge Constant d_31_ (pC/N)
Bare cellulose	5.3	6
Aligned cellulose	7.0	30
CEZOHN	5.0	145

**Table 4:** Electrode pattern effect on the actuator performance of cellulose EAPap actuators at 5 V, 90% RH, 25 °C [48].

Electrode Pattern	Max. Displ. (mm)	Resonance Frequency (Hz)	Electrical Power Consum. (mW)	Mechanical Power Output (μW)	Efficiency (%)
Rectangular (DCellR)	5.1 ± 0.11	7.5 ± 0.1	30 ± 1.2	3.6 ± 0.13	0.012 ± 0.001
Fishbone (DCellF)	4.8 ± 0.09	11.0 ± 0.1	28 ± 1.0	4.6 ± 0.15	0.013 ± 0.0012
Rectangular (CellR)	6.5 ± 0.12	5.5 ± 0.1	40 ± 1.3	4.3 ± 0.13	0.011 ± 0.0007
Fishbone (CellF)	5.6 ± 0.11	9.0 ± 0.1	28 ± 1.0	5.2 ± 0.16	0.019 ± 0.0017

**Table 5:** Analytical and experimental results of the V_p-p_, I_p-p_, and P_mean_ for partially covered cellulose-based piezoelectric energy harvester [79].

	V_p-p_ (mV) *	I_p-p_ (nA) **	P_mean_ (nW) ***
Experimental results	25.6	284	0.9071
Theoretical values	26.6	295	0.9831
Relative errors (%)	3.9	3.9	8.4

* Peak to peak voltage, ** peak to peak current, *** mean power output.

**Table 6:** Comparison of mechanical, electrical and piezoelectric properties of cellulose EAPap, zinc oxide nanocoated cellulose film (ZONCE), and polyvinylidene fluoride (PVDF) [27].

	ZONCE	Cellulose EAPap ^a^	PVDF
Young’s modulus (GPa)	3.5	5.0	2.7
Yield strength (MPa)	52.8	67.5	22.2
Tensile strength (MPa)	81.8	120.3	32.7
Dielectric constant (at 1 Hz)	21.3	16.0	13
Piezoelectric charge constant (pC/N)	93.5	26.5	20.0
Transparency (480 nm)	86.0	88.3	75.9

^a^ Results are for mechanical stretching one.

## References

[1] Spillman W., Sirkis J., Gardiner P. (1996). Smart materials and structures: What are they?. Smart Mater. Struct..

[2] Rogers C.A. (1993). Intelligent Material Systems—The Dawn of a New Materials Age.

[3] Curie J., Curie P. (1880). Development by pressure of polar electricity in hemihedral crystals with inclined faces. Bull. Soc. Min. Fr..

[4] Cox L.M., Killgore J.P., Li Z., Zhang Z., Hurley D.C., Xiao J., Ding Y. (2014). Morphing metal–polymer janus particles. Adv. Mater..

[5] Klemm D., Heublein B., Fink H.P., Bohn A. (2005). Cellulose: Fascinating biopolymer and sustainable raw material. Angew. Chem. Int. Ed..

[6] Cox L.M., Li Z., Sowan N., Nair D., Xiao J., Bowman C.N., Ding Y. (2014). Reconfigurable surface patterns on covalent adaptive network polymers using nanoimprint lithography. Polymer.

[7] Tadigadapa S., Mateti K. (2009). Piezoelectric mems sensors: State-of-the-art and perspectives. Meas. Sci. Technol..

[8] Bogue R. (2014). Smart materials: A review of capabilities and applications. Assembl. Autom..

[9] Quant M., Elizalde H., Flores A., Ramírez R., Orta P., Song G. (2009). A comprehensive model for piezoceramic actuators: Modelling, validation and application. Smart Mater. Struct..

[10] Fukada E. (2000). History and recent progress in piezoelectric polymers. IEEE Trans. Ultrason. Ferroelectr. Freq. Control.

[11] Naresh C., Bose P., Rao C. (2016). IOP Conference Series: Materials Science and Engineering. Shape Memory Alloys: A State of Art Review.

[12] Carpi F., De Rossi D., Kornbluh R., Pelrine R.E., Sommer-Larsen P. (2011). Dielectric Elastomers as Electromechanical Transducers: Fundamentals, Materials, Devices, Models and Applications of an Emerging Electroactive Polymer Technology.

[13] Choi Y., Cho J., Choi S., Wereley N. (2005). Constitutive models of electrorheological and magnetorheological fluids using viscometers. Smart Mater. Struct..

[14] Huang B., Koh B.-H., Kim H.S. (2014). Pca-based damage classification of delaminated smart composite structures using improved layerwise theory. Comput. Struct..

[15] Khan A., Lee H.S., Kim H.S. (2017). Analysis of sensor-debonding failure in active vibration control of smart composite plate. J. Intell. Mater. Syst. Struct..

[16] Xu J., Wang C., Li H., Zhang C., Hao J., Fan S. (2018). Health monitoring of bolted spherical joint connection based on active sensing technique using piezoceramic transducers. Sensors.

[17] Huang B., Kim H.S., Yoon G.H. (2015). Modeling of a partially debonded piezoelectric actuator in smart composite laminates. Smart Mater. Struct..

[18] Duan W.H., Wang Q., Quek S.T. (2010). Applications of piezoelectric materials in structural health monitoring and repair: Selected research examples. Materials.

[19] Xu X., Cao D., Yang H., He M. (2018). Application of piezoelectric transducer in energy harvesting in pavement. Int. J. Pavement Res. Technol..

[20] Kim H.S., Kim J.-H., Kim J. (2011). A review of piezoelectric energy harvesting based on vibration. Int. J. Precis. Eng. Manuf..

[21] Kim J., Yun S., Ounaies Z. (2006). Discovery of cellulose as a smart material. Macromolecules.

[22] Khan A., Abas Z., Kim H.S., Kim J. (2016). Recent progress on cellulose-based electro-active paper, its hybrid nanocomposites and applications. Sensors.

[23] Kim H.C., Mun S., Ko H.-U., Zhai L., Kafy A., Kim J. (2016). Renewable smart materials. Smart Mater. Struct..

[24] Kim J. (2009). Improvement of Piezoelectricity in Piezoelectric Paper Made with Cellulose.

[25] Abas Z., Kim H.S., Zhai L., Kim J., Kim J.H. (2014). Possibility of cellulose-based electro-active paper energy scavenging transducer. J. Nanosci. Nanotechnol..

[26] Yang C., Kim J.-H., Kim J.-H., Kim J., Kim H.S. (2009). Piezoelectricity of wet drawn cellulose electro-active paper. Sens. Actuators A Phys..

[27] Mun S., Ko H.-U., Zhai L., Min S.-K., Kim H.-C., Kim J. (2016). Enhanced electromechanical behavior of cellulose film by zinc oxide nanocoating and its vibration energy harvesting. Acta Mater..

[28] Yun G.-Y., Kim J.-H., Kim J. (2009). Dielectric and polarization behaviour of cellulose electro-active paper (EAPAP). J. Phys. D Appl. Phys..

[29] Kim J., Lee H., Kim H.S. (2010). Beam vibration control using cellulose-based electro-active paper sensor. Int. J. Precis. Eng. Manuf..

[30] Kim J., Jung W., Kim H.S. (2007). In-plane strain of electro-active paper under electric fields. Sens. Actuators A Phys..

[31] Kim H.S., Li Y., Kim J. (2008). Electro-mechanical behavior and direct piezoelectricity of cellulose electro-active paper. Sens. Actuators A Phys..

[32] Yun S., Kim J., Song C. (2007). Performance of electro-active paper actuators with thickness variation. Sens. Actuators A Phys..

[33] Standards Committee of the IEEE Ultrasonics, Ferroelectrics, and Frequency Control Society (1987). IEEE Standard on Piezoelectricity.

[34] Kim J., Lee H., Kim H.S., Kim J. (2010). Vibration sensor characteristics of piezoelectric electro-active paper. J. Intell. Mater. Syst. Struct..

[35] Abas Z., Yang D.H., Kim H.S., Kwak M.K., Kim J. (2015). Characterization of electro-active paper vibration sensor by impact testing and random excitation. Int. J. Appl. Mech..

[36] Lee S.-W., Kim J.-H., Kim J., Kim H.S. (2009). Characterization and sensor application of cellulose electro-active paper (EAPAP). Chin. Sci. Bull..

[37] Mun S., Zhai L., Min S.-K., Yun Y., Kim J. (2016). Flexible and transparent strain sensor made with silver nanowire–coated cellulose. J. Intell. Mater. Syst. Struct..

[38] Ko H.-U., Mun S., Min S.-K., Kim G.-W., Kim J. (2014). Fabrication of cellulose zno hybrid nanocomposite and its strain sensing behavior. Materials.

[39] Yun S., Kim J., Lee K.-S. (2010). Evaluation of cellulose electro-active paper made by tape casting and zone stretching methods. Int. J. Precis. Eng. Manuf..

[40] Macdiarmid A.G., Chiang J.-C., Halpern M., Huang W.-S., Mu S.-L., Nanaxakkara L., Wu S.W., Yaniger S.I. (1985). “Polyaniline”: Interconversion of metallic and insulating forms. Mol. Cryst. Liq. Cryst..

[41] Shahinpoor M., Bar-Cohen Y., Simpson J., Smith J. (1998). Ionic polymer-metal composites (IPMCs) as biomimetic sensors, actuators and artificial muscles—A review. Smart Mater. Struct..

[42] Re P. (1998). Electrostriction of polymer dielectrics with compliant electrodes as a means of actuation. Sens. Actuators A.

[43] Calvert P., Zengshe L. (1998). Freeform fabrication of hydrogels. Acta Mater..

[44] Kim J., Song C., Bae S.-H. (2005). Smart Structures and Materials 2005: Electroactive Polymer Actuators and Devices (EAPAD). Actuation Performance of Cellulose Based Electro-Active Papers.

[45] Kim J., Seo Y.B. (2002). Electro-active paper actuators. Smart Mater. Struct..

[46] Yun G.-Y., Yun K.-J., Kim J.-H., Kim J. (2011). Electrical and mechanical characterization of nanoscale-layered cellulose-based electro-active paper. J. Nanosci. Nanotechnol..

[47] Yun G.-Y., Kim J., Kim J.-H., Kim S.-Y. (2010). Fabrication and testing of cellulose eapap actuators for haptic application. Sens. Actuators A Phys..

[48] Ridley D.R., Williams F.R., Song K.D., Yun S., Kang K., Kim J. (2010). Effect of electrode pattern on the actuator performance of cellulose electro-active paper. J. Intell. Mater. Syst. Struct..

[49] Alici G., Higgins M.J. (2009). Normal stiffness calibration of microfabricated tri-layer conducting polymer actuators. Smart Mater. Struct..

[50] Kim J.-H., Yun K.-J., Kim J.-H., Kim J. (2009). Mechanical stretching effect on the actuator performance of cellulose electroactive paper. Smart Mater. Struct..

[51] Kim J., Yun S.R., Deshpande S. (2007). Synthesis, characterization and actuation behavior of polyaniline-coated electroactive paper actuators. Polym. Int..

[52] Yun G.-Y., Kim H.S., Kim J. (2008). Blocked force measurement of an electro-active paper actuator using a cantilevered force transducer. Smart Mater. Struct..

[53] Kim J., Kang Y., Yun S. (2007). Blocked force measurement of electro-active paper actuator by micro-balance. Sens. Actuators A Phys..

[54] Roundy S., Leland E.S., Baker J., Carleton E., Reilly E., Lai E., Otis B., Rabaey J.M., Sundararajan V., Wright P.K. (2005). Improving power output for vibration-based energy scavengers. IEEE Pervasive Comput..

[55] Harne R., Wang K. (2013). A review of the recent research on vibration energy harvesting via bistable systems. Smart Mater. Struct..

[56] Lee S., Youn B.D. (2011). A design and experimental verification methodology for an energy harvester skin structure. Smart Mater. Struct..

[57] Scholes G.D., Mirkovic T., Turner D.B., Fassioli F., Buchleitner A. (2012). Solar light harvesting by energy transfer: From ecology to coherence. Energy Environ. Sci..

[58] Guyomar D., Sebald G., Pruvost S., Lallart M., Khodayari A., Richard C. (2009). Energy harvesting from ambient vibrations and heat. J. Intell. Mater. Syst. Struct..

[59] Lee F.Y., Navid A., Pilon L. (2012). Pyroelectric waste heat energy harvesting using heat conduction. Appl. Therm. Eng..

[60] Krikidis I., Timotheou S., Sasaki S. (2012). Rf energy transfer for cooperative networks: Data relaying or energy harvesting?. IEEE Commun. Lett..

[61] Shrestha S., Noh S.-K., Choi D.-Y. (2013). Comparative study of antenna designs for rf energy harvesting. Int. J. Antennas Propag..

[62] Guigon R., Chaillout J.-J., Jager T., Despesse G. (2008). Harvesting raindrop energy: Experimental study. Smart Mater. Struct..

[63] Ong Z.-Z., Wong V.-K., Ho J.-H. (2016). Performance enhancement of a piezoelectric rain energy harvester. Sens. Actuators A Phys..

[64] Roundy S., Wright P.K., Rabaey J. (2003). A study of low level vibrations as a power source for wireless sensor nodes. Comput. Commun..

[65] Mitcheson P.D., Green T.C., Yeatman E.M., Holmes A.S. (2004). Architectures for vibration-driven micropower generators. J. Microelectromech. Syst..

[66] Bowen C., Kim H., Weaver P., Dunn S. (2014). Piezoelectric and ferroelectric materials and structures for energy harvesting applications. Energy Environ. Sci..

[67] Caliò R., Rongala U.B., Camboni D., Milazzo M., Stefanini C., De Petris G., Oddo C.M. (2014). Piezoelectric energy harvesting solutions. Sensors.

[68] Wang S., Lam K.H., Sun C.L., Kwok K.W., Chan H.L.W., Guo M.S., Zhao X.-Z. (2007). Energy harvesting with piezoelectric drum transducer. Appl. Phys. Lett..

[69] Motter D., Lavarda J.V., Dias F.A., Silva S.D. (2012). Vibration energy harvesting using piezoelectric transducer and non-controlled rectifiers circuits. J. Braz. Soc. Mech. Sci. Eng..

[70] Ahmed R., Mir F., Banerjee S. (2017). A review on energy harvesting approaches for renewable energies from ambient vibrations and acoustic waves using piezoelectricity. Smart Mater. Struct..

[71] Li H., Tian C., Deng Z.D. (2014). Energy harvesting from low frequency applications using piezoelectric materials. Appl. Phys. Rev..

[72] Jeon J.-H., Kang S.-P., Lee S., Oh I.-K. (2009). Novel biomimetic actuator based on speek and pvdf. Sens. Actuators B Chem..

[73] Vatansever D., Hadimani R., Shah T., Siores E. (2011). An investigation of energy harvesting from renewable sources with pvdf and pzt. Smart Mater. Struct..

[74] Wang P., Du H. (2015). ZnO thin film piezoelectric mems vibration energy harvesters with two piezoelectric elements for higher output performance. Rev. Sci. Instrum..

[75] Jung J.-H., Vadahanambi S., Oh I.-K. (2010). Electro-active nano-composite actuator based on fullerene-reinforced nafion. Compos. Sci. Technol..

[76] Jean-Mistral C., Basrour S., Chaillout J. (2010). Comparison of electroactive polymers for energy scavenging applications. Smart Mater. Struct..

[77] Yuan X., Changgeng S., Yan G., Zhenghong Z. (2016). Application Review of Dielectric Electroactive Polymers (Deaps) and Piezoelectric Materials for Vibration Energy Harvesting.

[78] Kim J., Wang N., Chen Y., Yun G.-Y. (2007). An electro-active paper actuator made with lithium chloride/cellulose films: Effects of glycerol content and film thickness. Smart Mater. Struct..

[79] Hosseini R., Hamedi M., Im J., Kim J., Dayou J. (2017). Analytical and experimental investigation of partially covered piezoelectric cantilever energy harvester. Int. J. Precis. Eng. Manuf..

[80] Abas Z., Kim H.S., Zhai L., Kim J. (2015). Experimental study of vibrational energy harvesting using electro-active paper. Int. J. Precis. Eng. Manuf..

[81] Abas Z., Kim H.S., Zhai L., Kim J., Kim J.-H. (2014). Electrode effects of a cellulose-based electro-active paper energy harvester. Smart Mater. Struct..

[82] Abas Z., Kim H.S., Zhai L., Kim J. (2015). Finite element analysis of vibration-driven electro-active paper energy harvester with experimental verification. Adv. Mech. Eng..

[83] Im J., Zhai L., Dayou J., Kim J.-W., Kim J. (2015). Nanosensors, Biosensors, and Info-Tech Sensors and Systems 2015. The Effects of Width Reduction on Cantilever Type Piezoelectric Energy Harvesters.

